# Paeonol attenuates inflammation by confining HMGB1 to the nucleus

**DOI:** 10.1111/jcmm.16319

**Published:** 2021-02-03

**Authors:** Jifei Miao, Jun Zhong, Jiao Lan, Sen Ye, Peng Ye, Siyan Li, Aijia You, Xianjie Chen, Xiaoyi Liu, Hui Li

**Affiliations:** ^1^ School of Chemical Biology and Biotechnology Peking University Shenzhen Graduate School Shenzhen China; ^2^ Research Center of Integrative Medicine School Basic Medical Sciences University of Chinese Medicine Guangzhou China; ^3^ Shenzhen Bao’an Traditional Chinese Medicine Hospital Shenzhen China; ^4^ School of Nursing Guangzhou University of Chinese Medicine Guangzhou China

**Keywords:** HMGB1, inflammation, NF‐κB, P65, paeonol

## Abstract

Inflammation is a biological process that exists in a large number of diseases. If the magnitude or duration of inflammation becomes uncontrolled, inflammation may cause pathological damage to the host. HMGB1 and NF‐κB have been shown to play pivotal roles in inflammation‐related diseases. New drugs aimed at inhibiting HMGB1 expression have become a key research focus. In the present study, we showed that paeonol (Pae), the main active component of *Paeonia suffruticosa*, decreases the expression of inflammatory cytokines and inhibits the translocation of HMGB1 induced by lipopolysaccharide (LPS). By constructing HMGB1‐overexpressing (HMGB1^+^) and HMGB1‐mutant (HMGB1^m^) RAW264.7 cells, we found that the nuclear HMGB1 could induce an LPS‐tolerant state in RAW264.7 cells and that paeonol had no influence on the expression of inflammatory cytokines in HMGB1^m^ RAW264.7 cells. In addition, the anti‐inflammatory property of paeonol was lost in HMGB1 conditional knockout mice, indicating that HMGB1 is a target of paeonol and a mediator through which paeonol exerts its anti‐inflammatory function. Additionally, we also found that HMGB1 and P50 competitively bound with P65, thus inactivating the NF‐κB pathway. Our research confirmed the anti‐inflammation property of paeonol and suggests that inhibiting the translocation of HMGB1 could be a new strategy for treating inflammation.

## 1 INTRODUCTION

Acute inflammation protects the host by eliminating hazards.^1^ Proper regulation and management of inflammation are fundamental to health.^2^ However, if the amplitude of an inflammatory response becomes uncontrolled, it will lead to numerous pathological condition, such as cardiovascular disease and sepsis, or even death.^3^ Inflammation is a complicated process that is related to numerous factors, including receptor for advanced glycation end products (RAGE), Toll‐like receptors (TLRs) and nuclear factor kappa B (NF‐κB).^4^, ^5^, ^6^ Among them, NF‐κB is the key regulator of inflammation. It regulates the expression of a large number of genes, especially those inflammation‐related genes, such as tumour necrosis factor‐α (TNF‐α), inducible nitric oxide synthase (iNOS) and interleukin‐1β (IL‐1β).^7^ Thus, manipulation of NF‐κB pathway has garnered extensive interest in the field of inflammation.

High‐mobility group box 1 (HMGB1) is widespread in the nucleus which maintains the stability of chromatin.^8^ In the late stage of inflammation, HMGB1 is highly acetylated and released from the nucleus.^9^, ^10^ Extranuclear HMGB1 activates NF‐κB signalling pathway and amplifies inflammatory response, acting as a lethal proinflammatory factor.^3^, ^8^, ^11^ Studies have proven that HMGB1 is the key factor in the mortality and multiorgan failure of septic shock.^12^, ^13^ Therapies using HMGB1 inhibitor have been proven to reduce mortality in sepsis models.^14^, ^15^ In addition, natural products with anti‐HMGB1 properties have also received attention from researchers.^16^, ^17^

**Figure 1:**
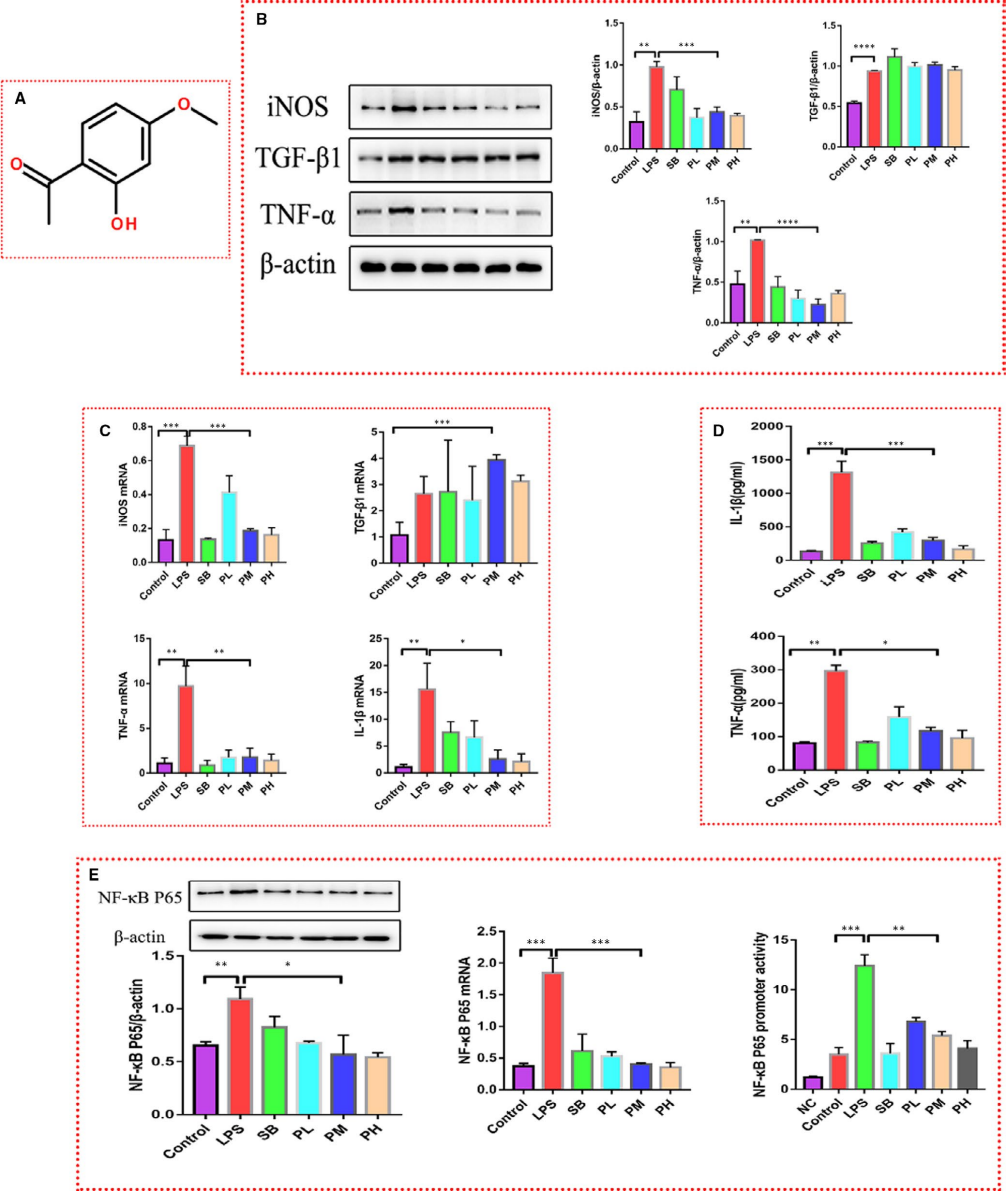
Paeonol attenuates the inflammation induced by LPS in RAW264.7 cells. A, The structure of paeonol is shown. B, iNOS, TNF‐α and TGF‐β1 levels in RAW264.7 cells treated with LPS, SB or paeonol were tested by using Western blotting. C, iNOS, TNF‐α, TGF‐β1 and IL‐1β levels were tested by using RT‐qPCR. D, IL‐1β and TNF‐α levels in cell culture supernatant were tested by using ELISA. E, Left: the protein level of P65 was tested by using Western blotting. Middle: the mRNA level of P65 was tested by using RT‐qPCR. Right: the promoter activity of P65 was tested by using a luciferase reporter assay. The bar graphs are representative of three independent experiments. *P < .05, ** *P* < .01 and *** *P* < .001

Paeonol (Pae; 2’‐hydroxy‐4’‐methoxyacetophenone) (Figure 1A), the major ingredient of *Paeonia suffruticosa*, has been proven to have anti‐inflammatory and antioxidative activities.^18^, ^19^ In our previous study, we found that paeonol could reduce mortality in septic shock rats and relieve acute lung injury induced by lipopolysaccharide (LPS).^20^ Furthermore, we also found that paeonol influences the localization of HMGB1 by up‐regulating histone deacetylase 3 (HDAC3) expression.^21^, ^22^ Therefore, we speculated that the mechanism underlying the anti‐inflammatory property of paeonol involves inhibiting the release of HMGB1 from the nucleus and NF‐κB may also take part in this process.

In the present study, HMGB1‐overexpressing (HMGB1^+^), HMGB1‐mutant (HMGB1^m^) RAW264.7 cells and HMGB1 conditional knockout mice were constructed to investigate how the nuclear HMGB1 affects inflammation. The study findings helped us to understand the mechanism detailing how paeonol exerts its anti‐inflammatory function. We show that paeonol inhibits the inflammatory response by suppressing the translocation of HMGB1 and the nuclear HMGB1 inhibits inflammation by binding to NF‐κB.

## 2 MATERIALS AND METHODS

### 2.1 Reagents

Paeonol, LPS and sodium butyrate (SB; purity > 99.9%) were obtained from Jianyang Co., Ltd. Primary antibodies specific for iNOS (ab178945), TGF‐β1 (ab179695), TNF‐α (ab1793), NF‐κB P65 (ab16502), HMGB1 (ab18256), β‐actin (ab6276), Lamin B1 (ab16048) and Flag (ab18230) were bought from Abcam. Secondary antibodies targeting mouse antibodies (ab6789 and ab150113) or rabbit antibodies (ab6721 and ab6939) were also bought from Abcam.

### 2.2 Cell culture

RAW 264.7 cell (Shanghai Institute of Cell Biology), HMGB1^+^ and HMGB1^m^‐RAW264.7 cells (The construction process was described previously^23^) were cultured in a culture medium containing Dulbecco's modified Eagle's medium (DMEM), foetal bovine serum (10%; FBS; Gibco; Thermo Fisher Scientific, Inc) and antibiotics (100 U/mL penicillin and 100 mg/mL streptomycin), which was placed in a humidified atmosphere of 5% CO_2_ at 37°C. When the cell confluency reached 70%, the culture medium was replaced with FBS‐free medium. Cells in LPS group were treated with LPS (0.2 μg/mL) for 24 h as an inflammatory cell model. Cells in paeonol groups were treated with both LPS (0.2 μg/mL) and paeonol (200 nmol/L, paeonol low‐dose (PL) group; 600 nmol/L, paeonol middle‐dose (PM) group; 1000 nmol/L, paeonol high‐dose (PH) group) for 24 h. Cells in SB group were treated with both LPS (0.2 μg/mL) and SB (10 mmol/L).

### 2.3 Animals and treatment

Eight‐week‐old male C57BL/6 mice (18‐20 g) were purchased from the Animal Laboratory Centre of Guangdong Province (Guangzhou, China). HMGB1 conditional knockout (Lyz‐2 cre, macrophage specific) mice were constructed by Cyagen Company. Mice were kept in a specific pathogen‐free (SPF) laboratory animal room at a constant temperature of 22°C and humidity of 50%‐70%, with a 12‐h light/dark cycle and free access to food and water. The mice were acclimatized to laboratory conditions for 3 d before experimentation. There were 6 mice included in each group. The mice in the LPS group were injected with LPS (15 mg/kg) and sacrificed after 24 h. The mice in the paeonol group were injected with both LPS (15 mg/kg) and paeonol (0.146 mg/kg) (the reason we choose this concentration is because that, in our another study, we found that paeonol at 0.146 mg/kg reverse the decrease of arteria blood pressure and high mortality of mice caused by LPS) and monitored for 24 h. Macrophages and tissues were obtained for subsequent Western blot, immunofluorescence and haematoxylin‐eosin (HE) analyses.

### 2.4 Western blot analysis

After treatment as described above, cells were washed 3 times with PBS and lysed with RIPA lysis buffer (1% protease inhibitor cocktail). Total protein was obtained from the supernatant after centrifugation (5000 rpm, 10 min), and cytosolic and nuclear fractions were extracted by using the Nuclear and Cytoplasmic Protein Extraction Kit (Sangon Biotech) according to the manufacturer's instructions. The protein concentration was determined by a BCA assay (Fdbio science). Proteins were boiled for 5 min and loaded into a 10%/8% Tris‐glycine gel (Beyotime). SDS‐PAGE was performed with running buffer at 80 V for 20 min for the spacer gel and 120 V for 60 min for the separation gel. Proteins were transferred onto a PVDF membrane (Millipore) by electroblotting. The membrane was blocked with milk and probed with a primary antibody (anti‐HMGB1, 1:1000; anti‐iNOS, 1:1000; anti‐TGF‐β1, 1:1000; anti‐TNF‐α, 1:1000; anti‐NFκB P65, 1:1000; anti‐Flag, 1:1000; anti‐β actin, 1:20 000; and anti‐lamin B1, 1:2000). Goat anti‐rabbit/mouse immunoglobulin G (IgG) conjugated with horseradish peroxidase (HRP) (Sino Biological, 1:2000) was used, followed by visualization using the enhanced chemiluminescence (ECL) method. βactin and lamin B1 were used as internal controls for cytoplasmic/total and nuclear protein analyses, respectively.

### 2.5 Enzyme‐linked immunosorbent assay (ELISA)

RAW264.7 cells were cultured and treated as described above. After treatment, the supernatant was collected. IL‐1β (Beyotime, PI301) and TNF‐α (Beyotime, PT512)‐specific ELISA kit were used. The experiment was conducted according to the operating instruction in the kit.

### 2.6 Luciferase reporter gene assay

A negative control plasmid was purchased from Gene Chem Company (Shanghai, China). A p65 promoter vector and an internal reference vector (pRL) were provided as a gift by Professor Hua Zichun (State Key Laboratory of Pharmaceutical Biotechnology, Nanjing University). RAW264.7 cells were transfected with these vectors. The cells were lysed after stimulation as described in subsection 2.2. The firefly luciferase and Renilla luciferase activities were measured at 465 nm (Renilla luciferase) and 560 nm (firefly luciferase) according to the protocol of a dual‐luciferase reporter assay kit (Promega Corporation), and relative fluorescence was determined by comparison with the negative control.

### 2.7 Reverse transcription‐polymerase chain reaction (RT‐qPCR)

Total RNA was extracted using TRIZOL® reagent (Thermo Fisher Scientific, Inc) according to the manufacturer's instructions. Reverse transcription and qPCR were conducted following the manufacturer's protocols for the Prime Script™ RT Reagent Kit and SYBR Premix EX Taq II Kit (Takara Biotechnology Co., Ltd.). The ViiA 7 Real‐time PCR System (Applied Biosystems; Thermo Fisher Scientific, Inc) was used with the following reaction conditions: 95°C for 10 s, 60°C for 60 s and 95°C for 15 s; 40 cycles. The 2−ΔΔCq method was used to analyse the expression of IL‐1β, TNF‐α, TGF‐β1, iNOS and P65. GAPDH was purchased from Sangon Biotech Co., Ltd. (cat. no. B661304). The primers were synthesized by Sangon Biotech Co., Ltd. (Table 1).

**Table 1:** Primer sequences

Gene	Species	Sequence (5’‐3’)
IL‐1β	Mouse	F: 5’‐TCGCAGCAGCACATCAACAAGAG‐3’ R: 5’‐TGCTCATGTCCTCATCCTGGAAGG‐3’
TNF‐α	Mouse	F: 5’‐ATGTCTCAGCCTCTTCTCATTC‐3’ R: 5’‐GCTTGTCACTCGAATTTTGAGA‐3’
TGF‐β1	Mouse	F: 5’‐CCAGATCCTGTCCAAACTAAGG‐3’ R: 5’‐CTCTTTAGCATAGTAGTCCGCT‐3’
iNOS	Mouse	F: 5’‐CACCTTGGAGTTCACCCAGT‐3’ R: 5’‐ACCACTCGTACTTGGGATGC‐3’
P65	Mouse	F: 5’‐TAGCACCTGATGGCTGACTG‐3’ R: 5’‐CGTTCCACCACATCTGTGTC‐3’

### 2.8 Immunofluorescence

Circular slides were placed in 24‐well plates, and cells were seeded on them at 10 000 cells/well and treated as described above. After treatment, the cells were fixed with paraformaldehyde, blocked with BSA and incubated with primary antibodies (anti‐HMGB1, 1:1000; anti‐Flag, 1:1000; and anti‐P65, 1:1000) overnight. The next day, the cells were incubated with fluorochromes (CY3, 1:1000; and FITC, 1:1000) after excess primary antibodies were removed. DAPI (1:1000, Beyotime) was used to stain nuclei. A laser scanning confocal microscope (Zeiss) was used for imaging.

### 2.9 Mammalian cell hybrid technology

HMGB1, P65 and P50 were cloned into the P‐act or P‐bind vector (accomplished by Beijing Genomics Institute). The vectors were transfected into 293T cells by using Lipofectamine® 3000 according to the manufacturer's instructions. Since 293T cells are more sensitive than RAW264.7 cells, many cells died after LPS stimulation. Therefore, we used TNF‐α stimulation. After paeonol treatment, cells were lysed and mixed with luciferase assay substrate to measure firefly luciferase activity. Fifteen seconds later, Stop&Glo substrate was added to test Renilla fluorescence (internal reference).

### 2.10 Transcriptomic analysis

RAW264.7 cells were cultured as described above and classified into 3 groups including the control, LPS and Paeonol group. Cells in Control group were treated with PBS, cells in LPS group were treated with LPS (0.2 μg/mL), and cells in Paeonol group were treated with both LPS (0.2 μg/mL) and paeonol (600 nmol/L). After 24h, the total RNA was extracted using TRIZOL® reagent (Thermo Fisher Scientific, Inc). mRNA was purified by using mRNA Capture Beads and fragmented by using Frag/Prime buffer. cDNA was synthesized using a two‐step process and purified with Hieff NGS™ DNA Selection Beads. After the DNA was amplified, gel electrophoresis was performed to test the length. Illumina Hiseq™ was used to read the sequence. Fast QC (GO.db, R Package) was used to visually evaluate the sequencing data quality of the samples. Trimmomatic was used to remove the low‐quality sequence. Differentially expressed genes were identified with qValue < 0.05 and |FoldChange|>2. Pathway enrichment analysis was performed by using igraph package in R. The transcriptomic analysis was conducted by Sangon Biotech company.

### 2.11 Molecular docking

The 3D structures of paeonol and chloroquine were retrieved from PubChem. The 3D structure of HMGB1 was downloaded from the RCSB PDB. The binding site of HMGB1 was determined according to a previous study.^24^ The molecular docking was performed with AutoDock 4.2.6. The energy grid was a 30 × 30 × 30 Å cube with a spacing of 0.375 Å between the grid points. The Lamarckian genetic algorithm (LGA) was used to optimize the conformations of compounds in the binding pocket. The parameters for the LGA were as follows: the number of individuals in the population, maximum number of energy evaluations, maximum number of generations, and rate of gene mutation were set as 150, 1.75 × 107, 2.7 × 104 and 0.02, respectively. Other parameters were set to default values.

### 2.12 Statistical analysis

All the data are shown as the mean ± standard error. Analyses were conducted with SPSS 21.0 software, and figures were plotted with GraphPad Prism 7.0 software. Differences among three or more groups were analysed by using one‐way ANOVA test. A value of *P* < .05 was considered statistically significant for all tests.

## 3 RESULTS

### 3.1 Paeonol inhibits inflammation induced by LPS in RAW264.7 cells

To confirm the anti‐inflammatory property of paeonol, LPS (0.2 μg/ml)‐stimulated RAW264.7 cells were used as an inflammatory cell model, while paeonol (200 nmol/L, PL group/600 nmol/L, PM group/1000 nmol/L, PH group) was used as the treatment. Given that the anti‐inflammatory effect of SB has been proven in many studies,^25^, ^26^ we used SB (10 mmol/L) as the positive control. TNF‐α, iNOS and TGF‐β1 were first evaluated (Figure 1B). After LPS stimulation, the levels of all these molecules were increased, indicating that LPS triggers an inflammatory response in RAW264.7 cells. Both SB and paeonol decreased the protein levels of TNF‐α and iNOS, which confirmed the anti‐inflammatory effect of paeonol. There were no differences among the three paeonol groups (PL, PM and PH, one‐way ANOVA test). However, SB and paeonol did not appear to influence TGF‐β1 levels, suggesting that they are specific to proinflammatory cytokines. RT‐qPCR and ELISA were also performed to test the transcriptional level and secreted level, respectively, of the abovementioned cytokines (Figure 1C, D), and the results showed a trend similar to that of the Western blot results. In addition, we also analysed the activity of the NF‐κB signalling pathway by assessing the protein and mRNA levels of P65 (Figure 1E‐left/middle). The P65 level was increased in the LPS group and decreased in the SB, PL, PM and PH groups. In addition, the promoter activity of P65 was suppressed by both SB and paeonol (Figure 1E‐right), indicating that the NF‐κB signalling pathway plays a role in the anti‐inflammatory function of paeonol.

### 3.2 Paeonol inhibits the translocation of HMGB1 from the nucleus to the cytoplasm

**Figure 2:**
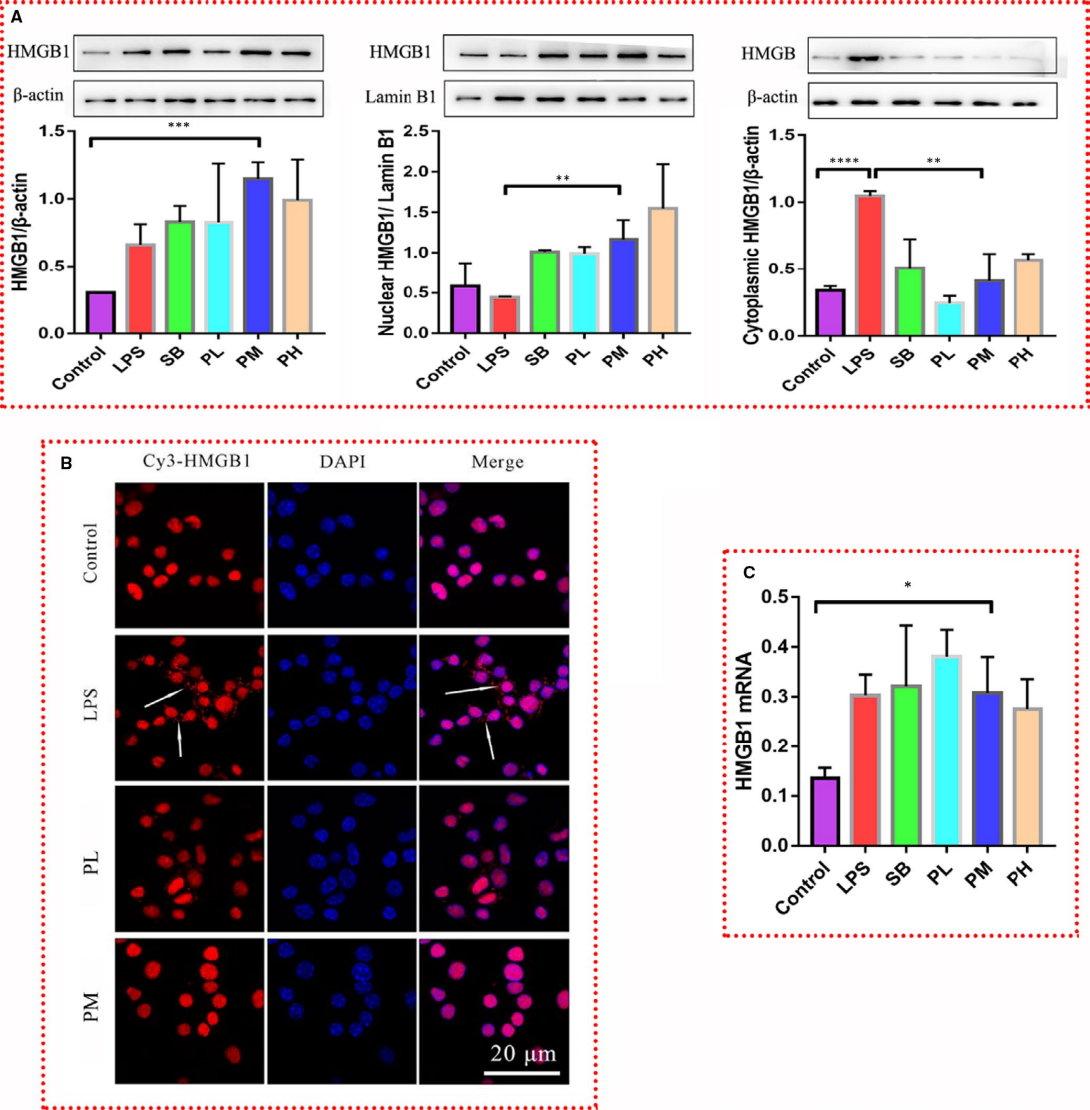
Paeonol inhibits the translocation of HMGB1 from the nucleus to the cytoplasm. A, Total protein (left), nuclear protein (middle) and cytoplasmic protein (right) were extracted. The level of HMGB1 in each fraction was tested by using Western blotting. B, HMGB1 was stained with Cy3 (red), and nuclei were stained with DAPI (blue). HMGB1 was distributed in the cytoplasm (indicated by the white arrows). C, The relative mRNA expression level of HMGB1 was tested by using RT‐qPCR. The bar graphs are representative of three independent experiments. *P < .05, ** *P* < .01 and *** *P* < .001

HMGB1 plays an important role in the development of inflammation. It is transported out of the nucleus in inflammatory environments, leading to an inflammatory storm via activation of the NF‐κB signalling pathway. Therefore, in addition to suppressing the expression of HMGB1, inhibiting the transport of HMGB1 out of the nucleus of immune cells is another viable anti‐inflammatory strategy. In our study, we first tested the level of HMGB1 in the total, nuclear and cytoplasmic protein fractions of RAW264.7 cells in each group (Figure 2A). In the total protein analysis, the HMGB1 level was increased after LPS stimulation, which agreed with the observed expression patterns of inflammatory cytokines (Figure 1B).

Interestingly, neither SB nor paeonol down‐regulated the expression of HMGB1, rather they up‐regulated this expression (Figure 2A‐left), suggesting that SB and paeonol do not attenuate inflammation by decreasing the HMGB1 level. However, RT‐qPCR results (Figure 2C) showed that there was no difference among the LPS, SB and paeonol groups (PL, PM and PH), indicating that paeonol has no influence on the transcription of HMGB1. In the nuclear protein fraction (Figure 2A‐middle), LPS decreased the level of HMGB1, while SB and paeonol increased it. The results for the cytoplasmic protein fraction (Figure 2A‐right) were opposite of those for the nuclear protein fraction. This phenomenon implies that paeonol inhibits the translocation of HMGB1 from the nucleus to the cytoplasm. Immunofluorescence was performed for further analysis (Figure 2B). In the control group, the position of HMGB1 (red) overlapped with that of the nucleus (blue), which indicates that HMGB1 is located in the nucleus under normal conditions. In the LPS group, HMGB1 was clearly transferred from the nucleus to the cytoplasm. This phenomenon was reversed in the PM group. The above results indicate that paeonol can inhibit the translocation of HMGB1 from the nucleus to the cytoplasm. This is the reason why paeonol can attenuate LPS‐induced inflammation. However, according to the results for the level of HMGB1 in the nucleus, the level of HMGB1 in the PM group was much higher than that in the LPS group. Therefore, we wondered whether there is a relationship between nuclear HMGB1 and the inflammatory response.

### 3.3 Mutation of the two nuclear localization sites in HMGB1 (from lysine to arginine) suppresses HMGB1 translocation from the nucleus to the cytoplasm

**Figure 3:**
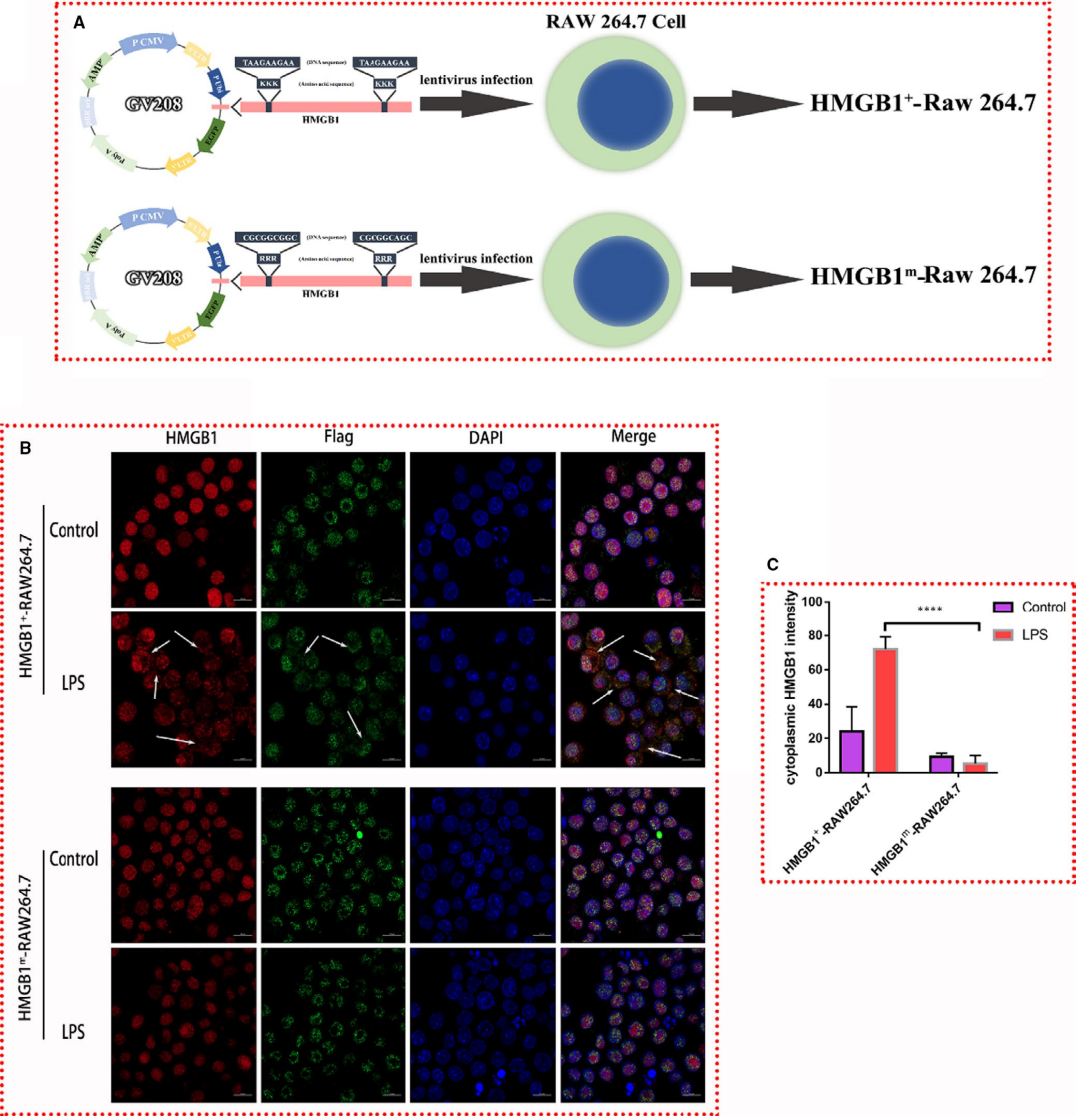
Construction of HMGB1^+^ RAW264.7 and HMGB1^m^ RAW264.7 cells. A, Diagram of HMGB1^+^ and HMGB1^m^ RAW264.7 cell construction. B, Endogenous HMGB1 was stained red, exogenous HMGB1 (Flag tagged, expressed from a plasmid) was stained green, and nuclei were stained blue. HMGB1 in the cytoplasm is indicated by white arrows. C, The intensity of cytoplasmic HMGB1 staining was measured with ImageJ software. *** *P* < .001

To answer the question posed above, we performed different interventions targeting HMGB1 in RAW264.7 cells. The HMGB1 gene was inserted into the GV208 plasmid, which is controlled by a Tet‐on gene expression system, and the plasmid was transfected into RAW264.7 cells (Figure 3A‐top). HMGB1^+^ RAW264.7 cells were selected by ampicillin screening. Additionally, we constructed HMGB1‐mutant RAW264.7 cells (HMGB1^m^ RAW264.7 cells) (Figure 3A‐bottom). The translocation of HMGB1 from the nucleus to the cytoplasm is based on the acetylation of the nuclear localization sites (NLSs) of HMGB1. Therefore, we changed the lysine residues localized in the two NLSs (27‐29 and 181‐183) to arginine residues. This method has been shown to effectively inhibit the translocation of HMGB1 from the nucleus to the cytoplasm.^27^

To better observe the localization of HMGB1 in HMGB1^m^ and HMGB1^+^ RAW264.7 cells, immunofluorescence was performed. Endogenous HMGB1 was stained red, exogenous HMGB1 (expressed by the plasmid, Flag tagged) was stained green, and the nucleus was stained blue (Figure 3B). Without LPS stimulation, both endogenous HMGB1 and exogenous HMGB1 were localized in the nucleus. After LPS stimulation, HMGB1 relocated to the cytoplasm in HMGB1^+^ RAW264.7 cells. However, there was no change in HMGB1^m^ RAW264.7 cells. To our surprise, the endogenous HMGB1 in HMGB1^m^ RAW264.7 cells also stayed in the nucleus after LPS stimulation, which we hypothesis to be due to the dimeric structure of HMGB1.^28^ All the above results convinced us that mutation of the NLSs could inhibit the translocation of HMGB1.

### 3.4 Paeonol attenuates inflammation in HMGB1^+^ RAW264.7 cells but not in HMGB1^m^ RAW264.7 cells

**Figure 4:**
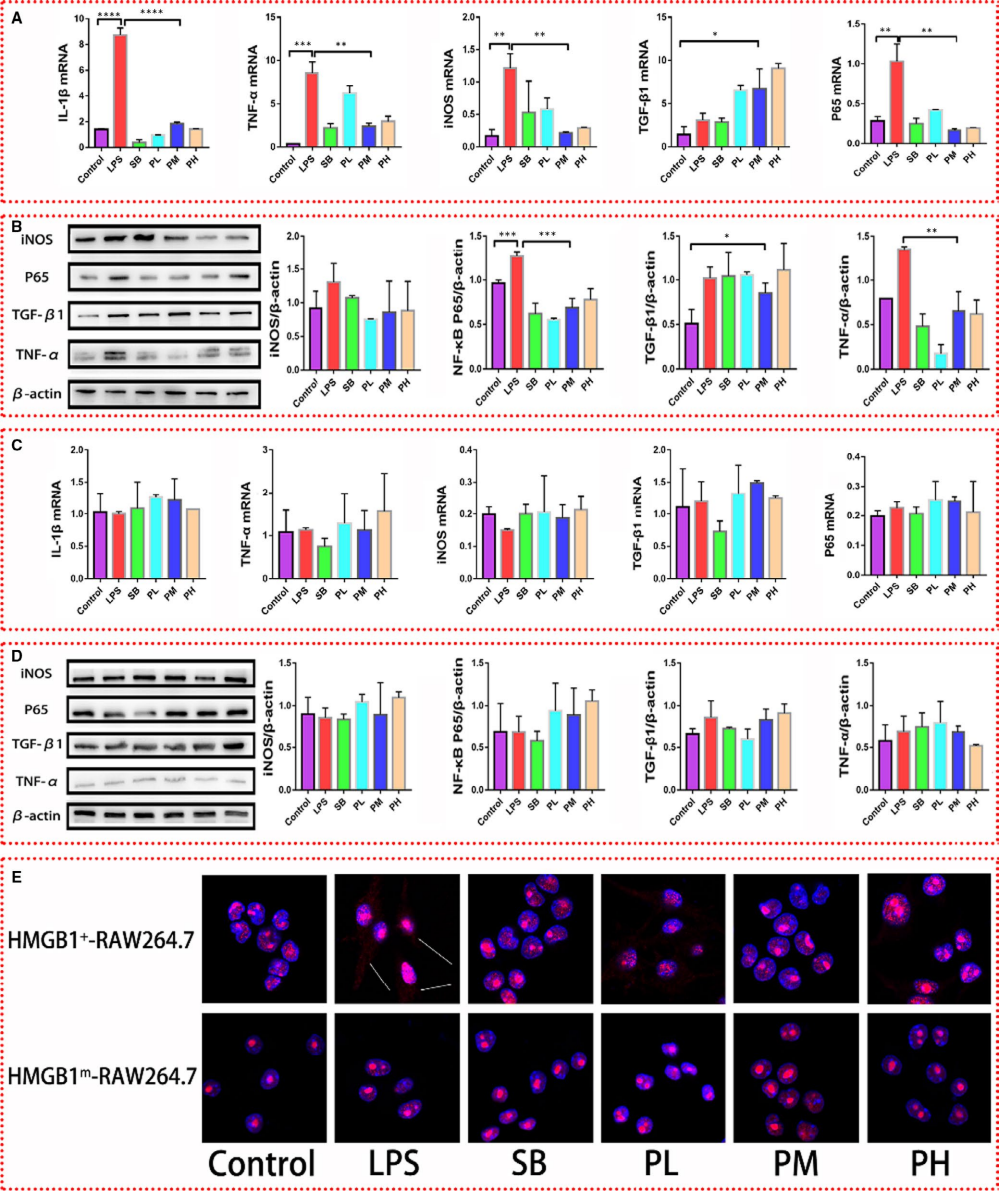
Influence of paeonol on the inflammatory response in HMGB1^+^ RAW264.7 and HMGB1^m^ RAW264.7 cells. A, The mRNA levels of IL‐1β, TNF‐α, iNOS, TGF‐β1 and P65 in HMGB1^+^ RAW264.7 cells were measured by using RT‐qPCR. B, The protein levels of IL‐1β, TNF‐α, iNOS, TGF‐β1 and P65 in HMGB1 + RAW264.7 cells were measured by using Western blotting. C, The relative mRNA expression levels of IL‐1β, TNF‐α, iNOS, TGF‐β1 and P65 in HMGB1m RAW264.7 cells were measured by using RT‐qPCR. D, The protein levels of IL‐1β, TNF‐α, iNOS, TGF‐β1 and P65 in HMGB1^m^ RAW264.7 cells were measured by using Western blotting. E, HMGB1 was stained red, and nuclei were stained blue. HMGB1 in the cytoplasm is indicated with white arrows. The bar graphs are representative of three independent experiments. *P < .05, ** *P* < .01 and *** *P* < .001

We proved that HMGB1 in HMGB1^m^ RAW264.7 cells is confined to the nucleus and that there is no limiting effect on the localization of HMGB1 in HMGB1^+^ RAW264.7 cells. Therefore, we tested the levels of inflammatory factors (IL‐1β, iNOS, P65, TNF‐α and TGF‐β1) in these two kinds of cells to investigate the influence of nuclear HMGB1 on inflammation. The treatment was the same as that described before (Figure 1). RT‐qPCR and Western blotting were performed, and the results for the inflammatory cytokines in HMGB1^+^ RAW264.7 cells (Figure 4A,B) were similar to those for the same cytokines in RAW264.7 cells (Figure 1). The mRNA levels of IL‐1β, TNF‐α, iNOS and P65 were higher in the LPS group than in the control group and lower in the SB and paeonol groups (PL, PM and PH) than in the LPS group (Figure 4A). The protein levels of P65 and TNF‐α (Figure 4B) exhibited the same pattern as the mRNA levels. Interestingly, the mRNA level of TGF‐β1 was much higher in the paeonol groups (PL, PM and PH) than in the LPS and SB groups (Figure 4A), while the protein level showed no difference (Figure 4B). The results for the levels of these inflammatory cytokines in HMGB1^m^ RAW264.7 cells were completely different from those in HMGB1^+^ RAW264.7 cells. HMGB1^m^ RAW264.7 cells seemed to tolerate LPS stimulation. The cytokines in all the groups showed no differences (Figure 4C,D). Immunofluorescence was performed to confirm the effect of paeonol on the localization of HMGB1 in HMGB1^m^ and HMGB1^+^ RAW264.7 cells. HMGB1 was stained red, and the nucleus was stained blue (Figure 4E). The HMGB1^+^ RAW264.7 cells showed results similar to those for RAW264.7 cells (Figure 1), but the HMGB1^m^ RAW264.7 cells did not show any changes. These results answered the question we posed above: nuclear HMGB1 could induce LPS tolerance in RAW264.7 cells and suppress the inflammation caused by LPS. This is another explanation for how paeonol attenuates inflammation.

### 3.5 Paeonol attenuates LPS‐induced inflammation in mice

**Figure 5:**
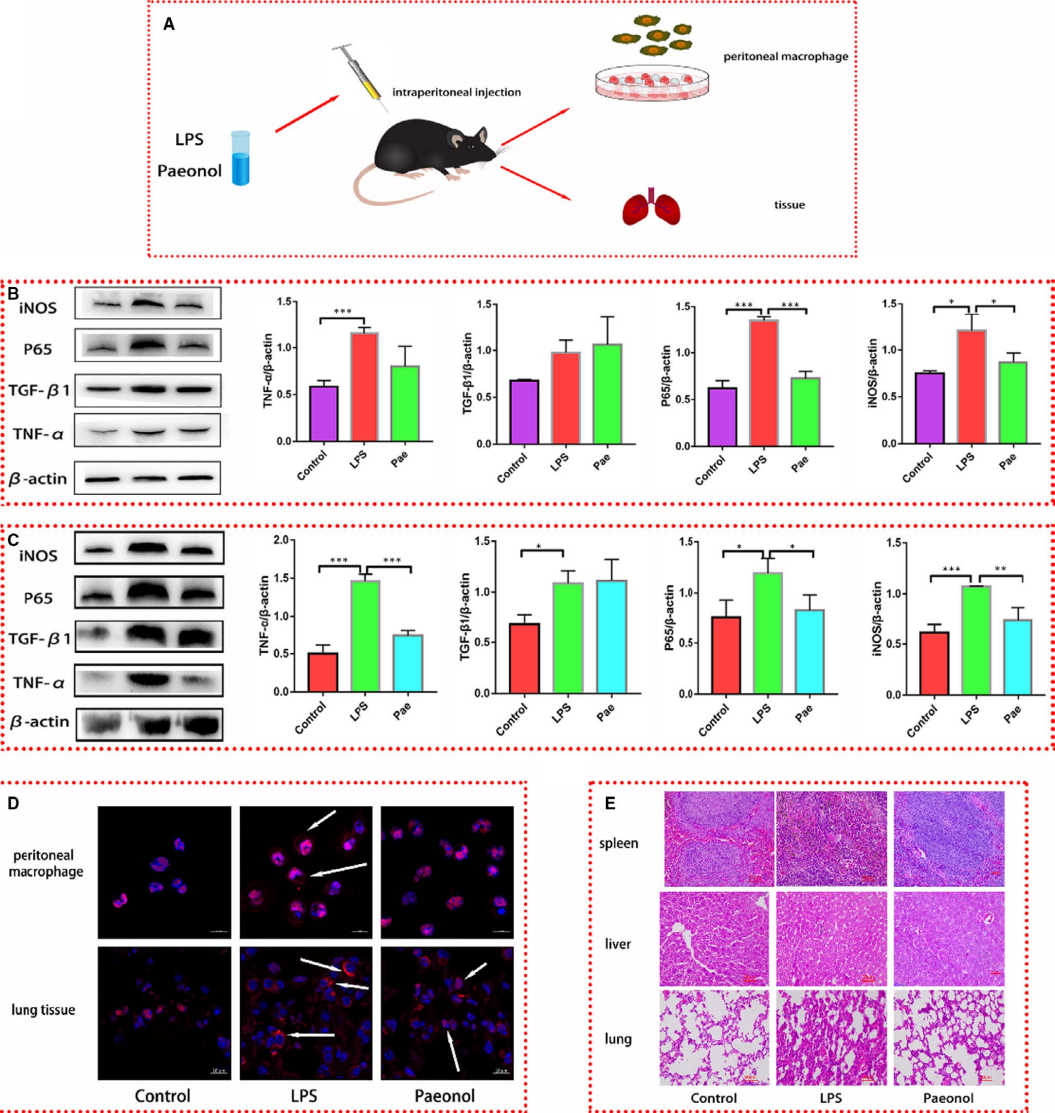
Paeonol attenuates the inflammatory response in vivo. A, A diagram of the animal experimental process is shown. B, The protein levels of iNOS, P65, TGF‐β1 and TNF‐α in peritoneal macrophages were tested by using Western blotting, n = 6. C, The protein levels of iNOS, P65, TGF‐β1 and TNF‐α in lung tissue were tested by using Western blotting, n = 6. D, HMGB1 in peritoneal macrophages and lung tissue was stained red. Nuclei were stained blue. HMGB1 in the cytoplasm is indicated by white arrows. D, HE staining was performed to observe the morphology of spleen, liver and lung tissues. *P < .05, ** *P* < .01 and *** *P* < .001

In addition to the in vitro study we performed, we also tested the anti‐inflammatory effect of paeonol in vivo. C57BL/6 mice injected with LPS were used as an inflammatory model (LPS group). Paeonol injection was used as the treatment (paeonol group). After 24 h of intervention, peritoneal macrophages and tissue were obtained for subsequent experiments (Figure 5A). We first tested the inflammatory cytokines in peritoneal macrophages and lung tissue. The results were the same as those of the in vitro study. TNF‐α, P65 and iNOS levels were all increased in the LPS group and decreased in the paeonol group, but the TGF‐β1 level did not differ between the LPS group and paeonol group (Figure 5B, C).

Immunofluorescence was performed to investigate whether paeonol has the same effect on HMGB1 in vivo. HMGB1 was stained red, and the nucleus was stained blue (Figure 5D). Similar to the results for RAW264.7 cells, HMGB1 in both peritoneal macrophages and lung tissue was localized in the nucleus in the control group, translocated into the cytoplasm in the LPS group, and located in the nucleus in the paeonol group (Figure 5D). To investigate whether paeonol has any protective function to limit the damage caused by LPS, we performed HE staining on three different organs, the spleen, liver and lungs. After LPS stimulation, the spleen and liver showed no change, but the lungs showed severe inflammatory oedema and hyperaemia. However, these phenomena were obviously limited after paeonol treatment. The above results confirmed that the in vivo anti‐inflammatory properties of paeonol are consistent with those observed in vitro.

### 3.6 Paeonol showed no protective effect on HMGB1 conditional knockout mice

**Figure 6:**
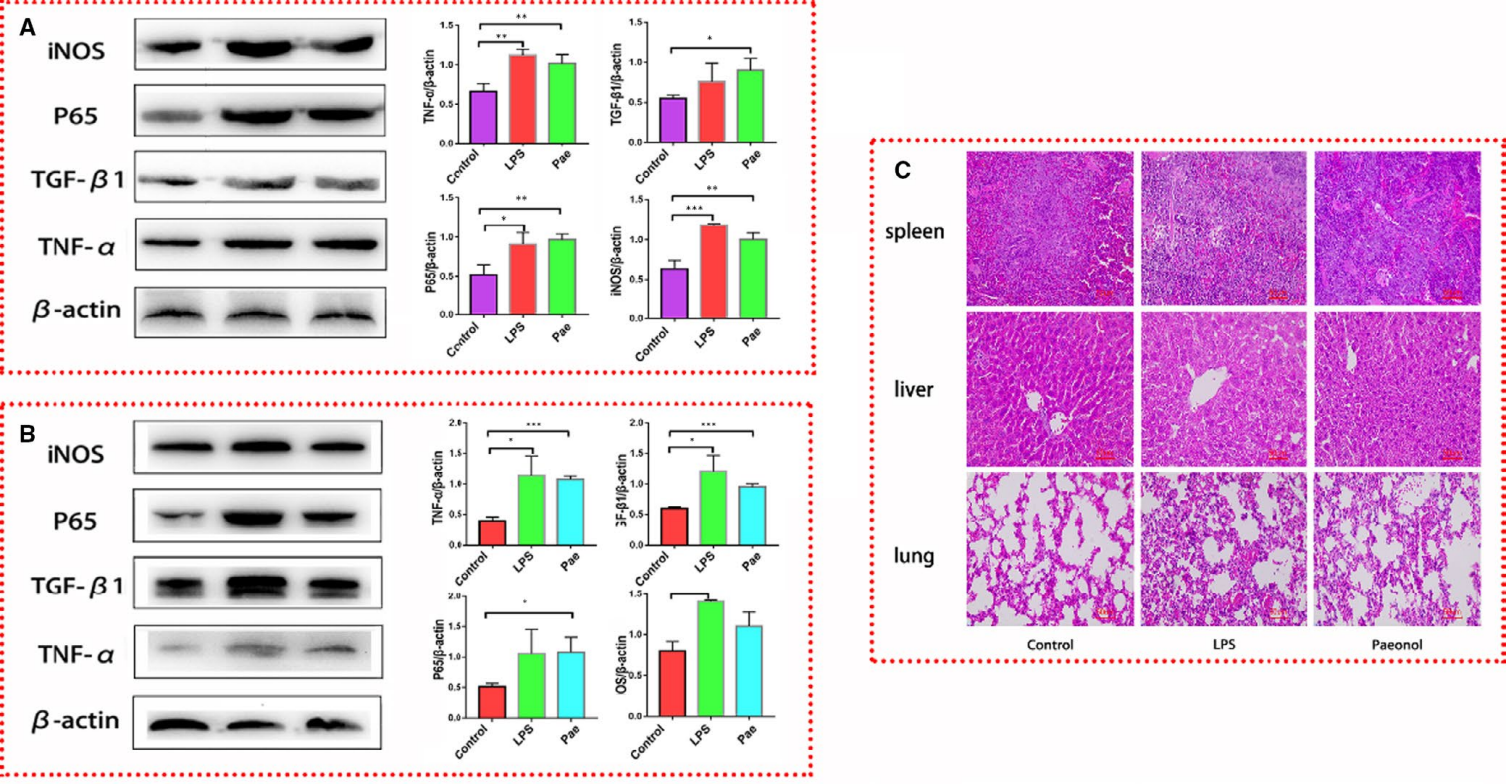
Paeonol has no effect on the inflammatory response in HMGB1 conditional knockout mice. A, The protein levels of iNOS, P65, TGF‐β1 and TNF‐α in peritoneal macrophages were tested by using Western blotting, n = 6. B, The protein levels of iNOS, P65, TGF‐β1 and TNF‐α in lung tissue were tested by using Western blotting, n = 6. C, HE staining was performed to observe the morphology of spleen, liver and lung tissues. *P < .05, ** *P* < .01 and *** *P* < .001

To verify the role of HMGB1 in the anti‐inflammatory function of paeonol, we constructed HMGB1 conditional knockout mice by using a Lox P‐Cre system. The knockout efficiency of HMGB1 was evaluated by using gene identification and Western blotting. We examined the level of HMGB1 in peritoneal macrophages, and the results showed that HMGB1 was completely knocked out (Figure S1). Conditional knockout mice were treated as before (Figure 5), and the inflammatory cytokines in peritoneal macrophages (Figure 6A) and lung tissue (Figure 6B) were evaluated. TNF‐α, iNOS, TGF‐β1 and P65 levels were all increased in the LPS group and paeonol group, but there was no difference between these groups. Paeonol appeared to not affect the levels of inflammatory cytokines. HE staining was also performed. Similarly, after LPS stimulation, the lung tissue was badly damaged. However, unlike the results described above, the damage was not limited in the paeonol group (Figure 6C). These results indicate that HMGB1 is a very important factor in the anti‐inflammatory function of paeonol. In the context of HMGB1 knockout, paeonol lost its protective anti‐inflammatory function in vivo.

### 3.7 Paeonol increased the binding rate between HMGB1 and P65

**Figure 7:**
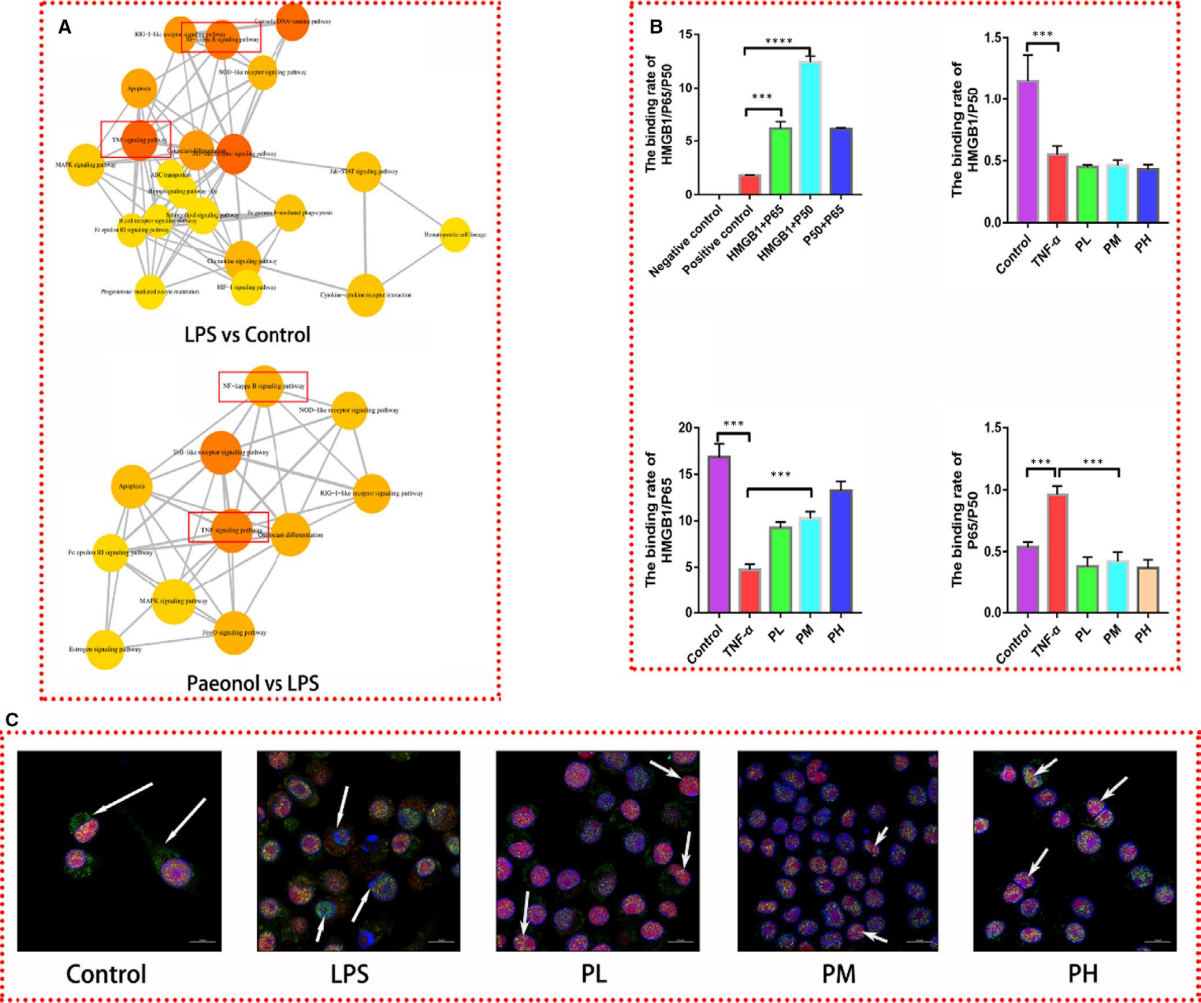
Paeonol promotes the colocalization of HMGB1 and P65. A, Pathway analysis of the transcriptome is shown. B, The binding rates among HMGB1/P65/P50 were analysed by using mammalian cell hybrid technology. C, HMGB1 was stained red, P65 was stained green, and nuclei were stained blue. P65 was found in the cytoplasm in the control group. In the LPS group, P65 was found in the nucleus, and HMGB1 was found in the cytoplasm. The complex of P65 and HMGB1 was identified in the PL, PM and PH groups. The bar graphs are representative of three independent experiments. *P < .05, ** *P* < .01 and *** *P* < .001

We proved that paeonol exerts its anti‐inflammatory function by confining HMGB1 to the nucleus. However, how HMGB1 works in the nucleus was still unclear. To identify potential targets, we treated RAW264.7 cells with LPS and paeonol and performed transcriptomic analysis. After analysing differentially expressed genes, as expected, the NF‐κB signalling pathway was identified to play a key role in this process (Figure 7A). NF‐κB regulates inflammation by activating the transcription of inflammatory genes, while HMGB1 is a DNA chaperone that participates in the transcription of many genes. Therefore, we believe that nuclear HMGB1 has a relationship with NF‐κB.

A mammalian two‐hybrid assay was performed to test the binding rates among HMGB1/P50/965 by using 293T cells (Figure 7B). Under normal conditions, HMGB1 could bind with both P50 and P65, with the binding rate between P50 and HMGB1 being the highest. We subsequently tested the binding rates among HMGB1/P50/P65 in an inflammatory environment and the influence of paeonol on these rates. Since LPS causes 293T cell death, we used TNF‐α as the inflammatory stimulus. In the TNF‐α group, the binding rates of both HMGB1/P65 and HMGB1/P50 were decreased, while that of P65/P50 was increased. The binding rate of HMGB1/P65 was increased in the paeonol groups (PL, PM and PH), and that of P50/P65 was decreased. These results indicated that paeonol promotes binding between HMGB1 and P65, thus reducing the aggregation of P65 and P50. To visualize these interactions, immunofluorescence was performed. In the control group, HMGB1 was localized in the nucleus, while P65 was dispersed in cells. In the LPS group, HMGB1 was secreted from the nucleus, while P65 was localized in the nucleus, which was opposite of the localization observed in the control group. In the paeonol groups (PL, PM and PH), both HMGB1 and P65 were localized in the nucleus and bound with each other. This phenomenon was most pronounced in the PH group. These results indicated that through binding with P65, nuclear HMGB1 inactivates the NF‐κB signalling pathway and inhibits the inflammatory response.

### 3.8 Computer simulation of the binding energy and combined conformation of paeonol and HMGB1

**Figure 8:**
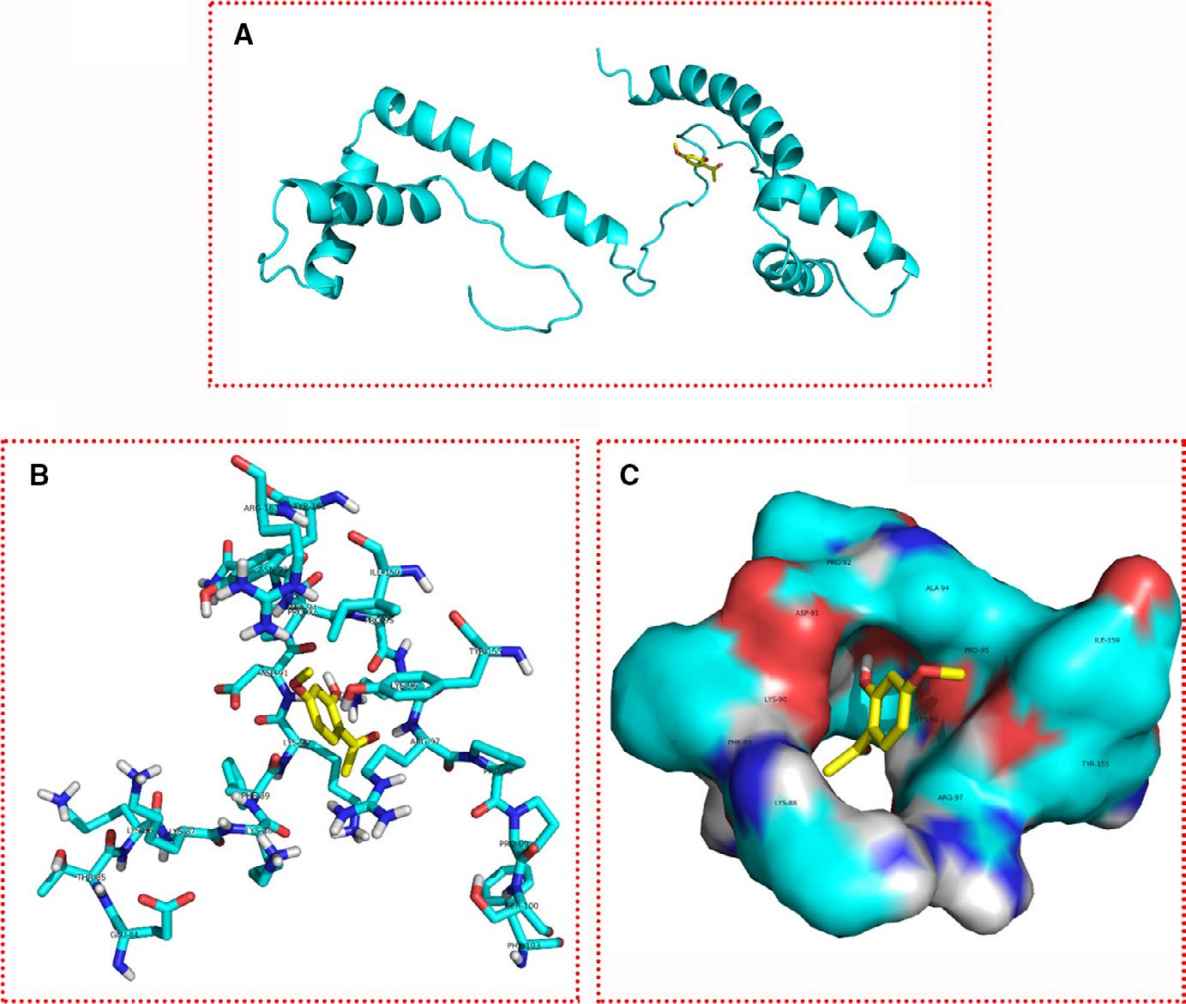
Combined conformation of paeonol and HMGB1. The yellow molecule is paeonol, and the cyan molecule is HMGB1

The 3D structures of HMGB1 and paeonol were obtained from the RCSB PDB and PubChem database (Figure 8), and Auto Dock was used to dock small molecules with target proteins. The binding energy of paeonol and HMGB1 was −5.77 kcal/mol. Paeonol bounds to a site in HMG box 2, the active domain of HMGB1, and might interfere with the LPS binding (amino acids 80‐96) and cytokine‐stimulating activity (amino acids 89‐108) of HMGB1. This result indicated that paeonol could directly interact with HMGB1 and regulate the synthesis and secretion of cytokines through HMGB1.

## 4 DISCUSSION

### 4.1 Nuclear HMGB1 induces LPS tolerance in macrophages

Inflammation is a complex pathological condition that is mainly caused by immune cells (such as macrophages, neutrophils and dendritic cells). Immune cells detect and respond to pathogens or inflammation‐triggered stimuli (such as enterocytes and cellular debris) through pattern recognition receptors. Among all these receptors, TLR‐4 is the major receptor related to the recognition of gram‐negative bacteria and LPS (a component of the cell wall of gram‐negative bacteria).^29^, ^30^ Mild inflammation is controllable, and it is necessary for the host to eliminate pathogens. However, once inflammation becomes uncontrolled, severe tissue damage and manifestations of pathological conditions, such as sepsis and shock, will occur.^31^ Blocking the development of uncontrolled inflammation has important clinical significance. LPS tolerance is a classic protective process to limit severe inflammation.^29^, ^32^, ^33^ Immune cells adopt a transient state that is tolerant to LPS after exposure to low concentrations of LPS.^34^ LPS binds to TLR‐4 and subsequently activates two pathways (myeloid differentiation primary‐response gene 88 (MyD88)‐dependent/independent pathways).^35^, ^36^ Both of these pathways will lead to the activation of NF‐κB and secretion of inflammatory cytokines. In this process, LPS cannot induce the secretion of inflammatory cytokines. Despite intense investigation in recent years, there is no consensus on how LPS tolerance is established.

In our study, we discovered that HMGB1 is a key factor for LPS tolerance, which is mainly related to its location in cells. After LPS stimulation, large quantities of inflammatory cytokines were secreted by HMGB1^+^ RAW264.7 cells but not HMGB1^m^ RAW264.7 cells, suggesting that HMGB1^m^ RAW264.7 cells are LPS tolerant. The difference between these two kinds of cells is the location of HMGB1 in the cells after LPS stimulation. This information implies that nuclear HMGB1 can induce an LPS‐tolerant state in RAW264.7 cells. It is well known that LPS causes the translocation of HMGB1 from the nucleus to the cytoplasm in the late stage of inflammation and that the LPS‐tolerant state is caused by stimulation with low concentrations of LPS. Therefore, one possible reason for the occurrence of LPS tolerance is that a low concentration of LPS can promote the transcription and translation but not the translocation of HMGB1, which causes accumulation of HMGB1 in the nucleus. There are 2 mechanisms by which nuclear HMGB1 induces the LPS‐tolerant state. First, according to our results, nuclear HMGB1 competitively binds P65, reducing the binding between P65 and P50 and thereby inhibiting the activation of the NF‐κB signalling pathway, which reduces the expression of inflammatory cytokines. Second, HMGB1 and the structural nucleosome linker H1 act as a component of the epigenetic complex. This complex promotes assembly of the NF‐κB repressor RelB and inhibits the transcription of TNF‐α and IL‐1β through binding with their promoters.^37^

Some researchers claim that preconditioning with HMGB1 induces LPS tolerance, and they believe that extracellular HMGB1 but not nuclear HMGB1 is involved in this tolerance.^38^, ^39^ Although they performed reverse validation using an anti‐HMGB1 antibody, they failed to verify differences between nuclear HMGB1 and extranuclear HMGB1. We know that extracellular HMGB1 is a proinflammatory factor and may be the inducer of systemic inflammatory cytokine storm through binding with RAGE/TLR‐4.^40^, ^41^ This property makes extracellular HMGB1 very similar to LPS. Therefore, pretreatment with HMGB1 should achieve results similar to those found with LPS pretreatment. Nuclear HMGB1 rather than extracellular HMGB1 is the inducer of LPS tolerance.

### 4.2 Paeonol alleviates inflammation by confining HMGB1 to the nucleus

As the most common endotoxin, LPS is usually used to investigate the mechanism of inflammation. In recent years, the mechanism of the inflammatory cascade induced by LPS has gradually been clarified. Through binding with TLRs and activation of the NF‐κB pathway, LPS promotes the expression of proinflammatory cytokines, such as TNF‐α and IL‐1β. In such an inflammatory environment, HMGB1 is released from cells in a noncanonical pattern and thought to be a fatal late inflammatory factor.^42^, ^43^ Inflammatory factors such as NF‐ B and TNF‐α, IL‐1β and HMGB1 are key targets of the molecular mechanism of the inflammatory response. Since HMGB1 may be the key toxic molecule responsible for late fatal endotoxin shock, inhibiting the secretion of HMGB1 can effectively reduce animal mortality.^44^ Therefore, in recent years, many studies have focused on drug development targeting HMGB1. To date, medicines such as anti‐HMGB1 antibodies, HMGB1 A box (an HMGB1 inhibitor)^45^ and ethyl pyruvate (EP)^46^ have been used in treatment studies. Natural products have also been investigated. Glycyrrhetinic acid can dock directly at the HMGB1 box and DNA‐binding pit to inhibit HMGB1 release.^47^ Carbenoxolone inhibits endotoxin‐induced HMGB1 release and prevents the up‐regulation of PKR activation and phosphorylation.^48^ Epigallocatechin gallate can stimulate the degradation of HMGB1 through autophagy.^49^ Tanshinone sodium IIA sulphonate can stimulate endocytosis by cells and promote the reuptake of HMGB1.^50^ In our study, we found that paeonol inhibits the activation of NF‐κB and significantly reduces the expression of TNF‐α and iNOS. Mechanistically, these effects occur through inhibition of the translocation of HMGB1 from the nucleus to the cytoplasm, which is completely different from the mechanisms of antibiotics, antibodies, inhibitors and natural products.

In addition to the mechanism (HMGB1 binding with NF‐κB) we found in this study, there is another possible mechanism. Previous studies have suggested that HMGB1 is a nonhistone intracellular structural protein that alters the stability of superhelical DNA but has no sequence specificity.^27^, ^51^ However, recent studies have found that dimeric HMGB1 can form specific protein or DNA domains, which may affect specific gene expression.^52^ Paeonol induces the accumulation of HMGB1 in the nucleus and thus may induce the formation of a large number of dimerized or polymerized HMGB1 molecules. These molecules may directly bind with inflammatory cytokine genes and inhibit their transcription. More experiments are needed to investigate this hypothesis. In addition, according to our AutoDock results, we found that HMGB1 directly binds with paeonol. Therefore, paeonol may also act as a transcription factor and influence the expression of inflammatory cytokines, but this hypothesis needs to be further investigated.

## 5 CONCLUSION

To our knowledge, numerous studies have demonstrated the anti‐inflammatory properties of paeonol.^53^, ^54^, ^55^ Our study is the first to show that paeonol can reduce inflammation by regulating HMGB1. Natural products are a great resource for identifying and selecting effective drugs. Our study provides a new direction for inflammation‐related disease treatment and drug development. The investigation of the molecular mechanism and the analysis of molecular docking lay the foundation for the development and synthesis of paeonol‐related drugs and paeonol derivatives.

## CONFLICTS OF INTEREST

The authors declare that they have no conflicts of interest.

## AUTHORS’ CONTRIBUTIONS

Jifei Miao performed most of the experimental work. Peng Ye and Jun Zhong cultured the cells. Aijia You, Xianjie Chen and Siyan Li bred the animals. Sen Ye analysed the data. Sen Ye and Jiao Lan helped write this article. Hui Li designed the research.

## ETHICAL APPROVAL

All animal care and experimental studies were approved by and conducted in accordance with the guidelines of the Animal Ethics Committee of Guangzhou University of Chinese Medicine. Mice were housed under SPF conditions at Guangzhou University of Chinese Medicine. All procedures were performed according to the Declaration of Helsinki.

## Supporting information

Fig S1
