# Supplementary figures and images for: Paeonol attenuates inflammation by confining HMGB1 to the nucleus
Source: J Cell Mol Med. 2021 Feb 3;25(6):2885–99. doi: 10.1111/jcmm.16319 (PMC7957162; doi:10.1111/jcmm.16319)

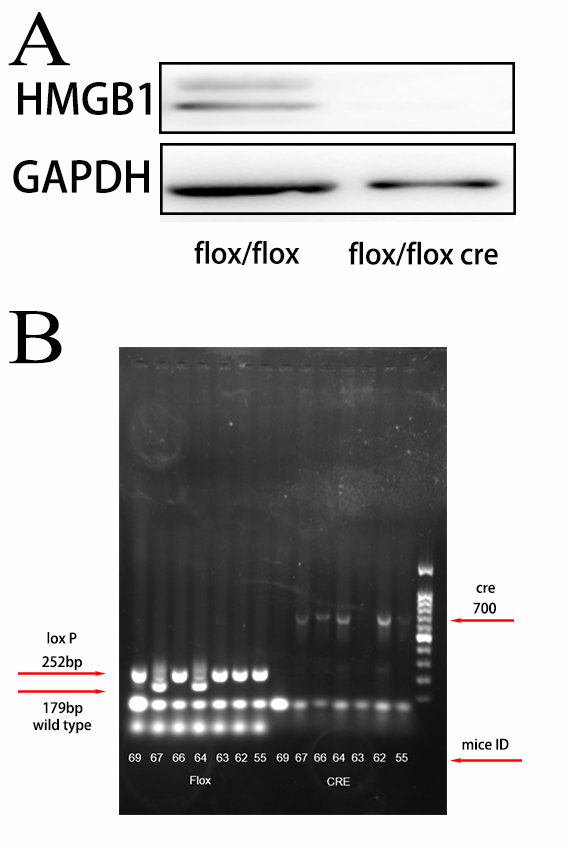

Supplement: Supplementary file 1 — Fig S1 [file JCMM-25-2885-s001.tif]
